# Cellular Responses during Morphological Transformation in *Azospirillum brasilense* and Its *flcA* Knockout Mutant

**DOI:** 10.1371/journal.pone.0114435

**Published:** 2014-12-12

**Authors:** Xingsheng Hou, Mary McMillan, Joëlle V. F. Coumans, Anne Poljak, Mark J. Raftery, Lily Pereg

**Affiliations:** 1 School of Science and Technology, University of New England, Armidale, New South Wales, Australia; 2 Department of Microbiology and Immunology, Shanxi Medical University, Taiyuan, Shanxi, China; 3 School of Rural Medicine, University of New England, Armidale, New South Wales, Australia; 4 Bioanalytical Mass Spectrometry Facility, Analytical Centre, University of New South Wales, Sydney, New South Wales, Australia; 5 The School of Medical Sciences, University of New South Wales, Sydney, New South Wales, Australia; Hans-Knoell-Institute (HKI), Germany

## Abstract

FlcA is a response regulator controlling flocculation and the morphological transformation of *Azospirillum* cells from vegetative to cyst-like forms. To understand the cellular responses of *Azospirillum* to conditions that cause morphological transformation, proteins differentially expressed under flocculation conditions in *A. brasilense* Sp7 and its *flcA* knockout mutant were investigated. Comparison of 2-DE protein profiles of wild-type (Sp7) and a *flcA* deletion mutant (Sp7-flcAΔ) revealed a total of 33 differentially expressed 2-DE gel spots, with 22 of these spots confidently separated to allow protein identification. Analysis of these spots by liquid chromatography-tandem mass spectrometry (LC-MS/MS) and MASCOT database searching identified 48 proteins (≥10% emPAI in each spot). The functional characteristics of these proteins included carbon metabolism (beta-ketothiolase and citrate synthase), nitrogen metabolism (Glutamine synthetase and nitric oxide synthase), stress tolerance (superoxide dismutase, Alkyl hydroperoxidase and ATP-dependent Clp protease proteolytic subunit) and morphological transformation (transducer coupling protein). The observed differences between Sp7 wild-type and *flcA* ^−^ strains enhance our understanding of the morphological transformation process and help to explain previous phenotypical observations. This work is a step forward in connecting the *Azospirillum* phenome and genome.

## Introduction

*Azospirillum brasilense* Sp7 is a Gram-negative, free-living bacterium that associates with plant roots, excretes plant hormones and fixes nitrogen in the rhizosphere [1], [2]. *A. brasilense* strains can change their metabolic activities and their form (morphologically transform) in response to environmental changes. Under stress conditions or nutrient limited conditions *A. brasilense* strains convert into ovoid, less motile, encapsulated cyst-like forms [3], [4]. Under the above conditions, *Azospirillum* cells develop a matrix of exopolysaccharides (EPS) and form macroscopic aggregates with accumulation of poly-β-hydroxybutyrate (PHB) granules within the cells [2], [5]–[9]. *Azospirillum* cells attached to plant roots often take a swollen, round shape resembling cyst-like forms, however, interestingly, they have been shown to be loaded with ribosomes and therefore metabolically active in the rhizosphere [10].

The protein FlcA, a 215 amino acid protein, belongs to the LuxR family of transcriptional regulators. It controls the morphological transformation process of *A. brasilense* cells from vegetative to cyst-like forms, both in culture and in association with plants [6], [11]. In contrast to the wild type strain Sp7, Tn5-induced *flcA* ^−^ mutants do not change from motile, vibroid cells into less motile, cyst-like forms. They also lack components of the EPSs, do not flocculate, do not bind Congo-Red and colonize the root surface to a lesser degree than wild-type Sp7 [6]–[8].

To date there is little known regarding the target genes controlled by FlcA and other cell aggregation-related genes [12]. Therefore, we used a proteomic approach to gain insight into the molecular basis of cell aggregation and morphological transformation in *A. brasilense* involving FlcA. Two-dimensional gel electrophoresis (2-DE) was used to reveal proteins differentially expressed between the wild type strain Sp7 and the *flcA* in-frame knock out strain, Sp7-flcAΔ. The differentially expressed proteins were then analyzed by LC-MS/MS and identified by MASCOT database searching. Quantitative Reverse Transcriptase Polymerase Chain Reaction (qRT-PCR) was carried out to further analyze expression of genes encoding the differentially expressed proteins at the mRNA level. The data presented here provide the molecular evidence for FlcA involvement in stress tolerance, carbohydrate metabolism, morphological transformation and nitrogen fixation.

## Materials and Methods

### Bacterial strains and culture conditions

The bacterial strains and plasmids used in this work are listed in Table 1. *A. brasilense* strains were grown aerobically at 30°C, 180 rpm, in nutrient broth medium (NB; Difco) or nitrogen-free medium (NFB) [13]. *E. coli* strains were grown at 37°C in Luria-Bertani medium (LB; Difco). Antibiotics and Congo-Red were added at the following final concentrations when required: 100 µg/mL ampicillin (Amp), 20 µg/mL kanamycin (Kan), 5 µg/mL tetracycline (Tet) and 40 µg/mL Congo-Red.

**Table 1:** Strains and plasmids used in this study.

Strain	Characteristics	Reference
***E. coli***		
DH5α	F-, SupE44Δ lacU169 (φ80 lacZΔM15) hsdR17 recA1 endA1 gyrA96 thi-1 relA1	[85]
JM109	endA1, recA1, gyrA96, thi, hsdR17 (rk–, mk+), relA1, supE44, Δ(lac-proAB),	[86]
S17.1	recA thi pro hsdRM+RP4: 2-Tet:Mu:KmTn7 TpR SmR	[6]
BMH8117	F− (lac-proAB) thi, gyrA (NalR), supE, λ−	[87]
***A. brasilense***		
Sp7	Wild type	[6]
Sp72001	Tn5 induced *flcA* mutant of Sp7; Km^r^ CR^−^ Floc^−^	[6]
Sp7-flcAΔ	In-frame deletion of *flcA* in Sp7; Tet^r^ CR^−^ Floc^−^	This work
**Plasmid**		
pBR322	ColE1, Amp, Tet	[88]
pAB2000	pSUP202 derivative, containing *flcA* gene on a 9 kb HindIII fragment	[6]
pAB2062b	pTZ18R derivative, contains *flcA* on a 4.6-kb BamHI-BglII fragment	[6]
pSUP202	ColE1, Cat, Tet, Bla, Rop	[15]
pBRd322	D322 derivative, contains a 1kb NcoI-NotI fragment from downstream of *flcA*	This work
pSUP-flcAΔ	pSup202 derivative, contains a 1.5kb PstI-StuI fragment from upstream of flcA, tet, and a 1kb NcoI-NotI fragment from downstream of flcA	This work
pAB2053	pLA29.17 derivative, contains *flcA* on a 4.6-kb BamHI-BglII fragment	[6]
pLA-lacZ	pLA29.17 derivative, Tet^r^, Km^r^, lacZ-kan constitutive fusion	[6]
D322	pBR322 derivative, delete MscI-BspEI fragment, instead of a NcoI, NotI, XhoI linker	This work

Amp^r^, Km^r^, Tet^r^ indicate resistance to ampicillin, kanamycin, and tetracycline, respectively; CR, Congo red binding; Floc, flocculation.

### Knockout of flcA in Azospirillum brasilense Sp7

All molecular manipulations were performed by conventional techniques [14] or instructions provided by the manufacturers. A 1.5 kb *Pst*I-*Stu*I fragment containing the upstream sequence of Sp7 *flcA* from pAB2000 [6], a 1.5 kb *Eco*RI-*Msc*I fragment containing the tetracycline gene from pBR322 and a 1.0 kb *Nco*I-*Not*I fragment containing the downstream sequence of *flcA* from pAB2000 were sub-cloned in the suicide plasmid pSUP202 [15], so that the *flcA* sequences were flanking the tetracycline resistance gene. The final plasmid construct, named pSUP-flcAΔ, was transformed into *E. coli* donor strain S17.1 for conjugation with A. *brasilense* Sp7 as described by Pereg Gerk et al. [6]. The *flcA* knock-out strain, Sp7-flcAΔ and Sp72001 [6] were analyzed by Southern Blot hybridization, digesting the genomic DNA with *Hind*III and using amplified *flcA* or Tetracycline resistance genes as probes. In addition, PCR amplification of genomic DNA and sequencing with primers derived from sequences up- and down-stream of *flcA* (FlcA-up, AACTCTCCTGACCGCAAATG; FlcA-down, AACCTTCTGGACCCTCGGAC; or Tn5-IR, ATGGTGGCGATAACTCAAAGA) were performed. Complementation of *flcA* in the knock out strain was performed by conjugation of Sp7-flcAΔ with S17.1 [pAB2053] donor strain and selection of *flcA*-complemented strain (Sp7-flcAΔ [pAB2053]) on NFB supplemented with kanamycin, tetracycline and Congo-Red [6].

Primers for amplifying Southern-Blot probes were:

ProbeFlcA-up (CGTCTTCTGGAGCAGCTTCACG) and ProbeFlcA-down (ATCACCGCCTGGGTGCGGTTC) for *flcA* amplification from plasmid pAB2062B (Table 2); ProbeTet-up (AATCTAACAATGCGC) and ProbeTet-down (TGTCCTACGAGTTGC) for tetracycline gene amplification from plasmid pBRd322 (Table 2). Oligonucleotides were synthesized by Geneworks Pty. Ltd. (Hindmarsh, Australia). DNA sequencing was carried out by Supamac (Sydney, Australia).

**Table 2:** qRT-PCR primer sets used in this study.

Gene	Forward Primer (5′-3′)	Reverse Primer (5′-3′)	Amplicon size (BP)
*AhpC*	CAAGTGGTCGGTCGTCTTCT	TAGATCTCAACGCCGAGCTT	114
*AtpD*	AGCTGTCGGAAGAGGACAAG	CTTGAAGCCCTTGATGGTGT	143
*CheW*	GCCTCTCCAACGATGACTTC	GACCGTTCAGGCGATAGATG	90
*ClpP*	CTGTTCCTGGAATCGGAAAA	GGATGTACTGCATGGTGTCG	109
*GalU*	ACCACAAGAGAACCCCAATG	GGTGACGAACACGAAATCCT	191
*GlnA*	CCGAGTTCTTCGTCTTCGAC	CAGGTTGCCGTCCTCATAGT	119
*GloA*	CCTGGAACTGACCCACAACT	ACGCTGTGAGACCGCTTACT	241
*GltA*	AGTCGGCGATCACCTACATC	TGGCAGACCTCCAGGTAATC	103
*GlyA*	GGAGATCGCCAAGAAGATCA	GCTCTTGGCGTAGGTCTTGA	133
*GyrA*	TCACCGACGAAGAGTTGATG	CTCTTCGATCTCGGTCTTGG	143
*LivK*	CTTGGCGATGATCGGACTAT	GTTTCGTTGGCTCCCTGTTA	120
*LpxC*	GACGAGGTGATGAACGAAGG	GGAAGTGACCGACGAACATT	115
*NarK*	ATCAACGGGTGGCTCTACAT	ATTGTGCCTTCACCAGCATT	82
*NarL1*	GAGGCTTCTCGACGATTCAC	ACCAGATCGAGCAGGATGAG	114
*Nos*	AACTACGACGCCATCATCCT	CTGGAAGCTGTAGGGCAGAC	240
*PhbA*	AGGACATCGAGGATGTGGTC	CAATAGCGGTTGATCGTGGT	130
*SodA*	CATCAGGCTTACGTCGACAA	CCAGAACATGCTGTGATTCG	165

### Phenotype confirmation of *flcA* knock-out mutant, Sp7-flcAΔ

#### Examination of flocculation and Congo-Red binding

Flocculation tests were performed as previously described [13]. Cultures were first grown in NB medium to an A_600_ of 0.8–0.9 and the cells were harvested by centrifugation at 10,000×g for 1 min. The pellet was washed in minimal medium [13] and then used to inoculate 10 mL of flocculation medium (minimal medium supplemented with 8 mM fructose and 0.5 mM KNO_3_) in a 50 ml flask, to an A_600_ of 0.3–0.4. The flasks were incubated with shaking at 200 rpm, 28°C, and checked periodically for flocculation, which took place within 3–4 hours in wild-type Sp7. Flocculation was observed visually and by stereomicroscope (Nikon SMZ800). The NB cultures of *Azospirillum* strains were also used for a loop spread on solid minimal lactate medium containing 40 µg/mL Congo-Red [6] incubated at 30°C for 3–4 days, and resultant colonies were examined for color and morphology by stereomicroscopy (Nikon SMZ800).

#### Colonization of wheat roots

The plasmid pLA-lacZ [6], containing a constitutively expressed *lacZ* gene cassette, was transferred by conjugation from *E. coli* S17.1 to both *A. brasilense* strains Sp7 and Sp7-flcAΔ. The above *Azospirillum* pLA-lacZ-containing strains were used to inoculate wheat root seedlings as previously described [6]. Ten days after inoculation, the wheat roots were sectioned into 2 cm long segments, and stained with X-gal as previously described [11]. The root sections were examined by light microscopy (Nikon YS2-H) and photographed.

#### Protein extraction

*A. brasilense* strains were grown in NB media at 30°C with shaking and bacteria in logarithmic phase (A_600_ 0.5–0.8) were collected by centrifugation. For flocculation conditions, bacteria were collected by centrifugation after 3–4 hours shaking at 30°C in flocculation medium [6]. About 50 mg cells were lysed and homogenized using a mini beadbeater containing a mixture of glass beads (G4649 and G1277, Sigma) and lysis buffer (30% sucrose, 0.1 M Tris pH 8.0, 2 mM PMSF, 1% DTT, 100 mM KCl, 5 mM EDTA) 4 times for 30 sec. Protein extraction was performed from the lysates by phenol extraction [16] and proteins were precipitated overnight using 5 volumes of 0.1 M ammonium acetate in methanol at −20°C. The protein solution was subsequently centrifuged at 6,000 g for 10 min and the pellet rinsed twice with 0.1 M ammonium acetate in methanol, three times with cold methanol and once with 80% acetone. The protein pellet was then cold-dried using a vacuum freeze dryer for 4 hours, then dissolved in IEF buffer (7 M Urea, 2 M Thiourea, 4% CHAPS, 0.5% IPG buffer pH 3–10, 1% DTT, 0.2% Coomassie Brilliant Blue). The protein concentration was determined using the 2D Quant Kit (GE Healthcare Life Science, Australia).

#### Two-dimensional gel electrophoresis and image analysis

The first dimension was carried by cup loading onto a rehydrated 17 cm IPG strip, pH 5–8 (Bio-Rad) (250 µg protein per analytical gel and 450 µg protein per preparative gel) and focused using the IPGphor isoelectric focusing unit (GE Healthcare Life Science) (20°C with current limit of 50 µA/strip) to a total volt-hour product of 32 kVh (analytical gels) or 45 kVh (preparative gels). Prior to running the second dimension, the strips were first equilibrated in DTT and then in iodoacetamide [17]. The second dimension was performed on lab-cast 12% SDS-PAGE using the PROTEAN II system (Bio-Rad). Proteins were visualized by Blue silver staining for analytical gels [18] and Coomassie blue R-250 [14] for preparative gels. Gel images from three technical replicates and two biological replicates (total of six gels for each strain), were taken using the infinity imaging system (Vilber Lourmat, France), and analyzed using the PDquest advanced 2-D analysis software (Bio-Rad). Numbers of biological and technical replicates that should be used in this kind of experimental design have not been standardized in the published literature. However the numbers we have used are typical of published work using a similar approach [19]–[22].Spots that had at least a 2-fold change in their expression level and found by ANOVA (Excel) to be statistically significant (*P*<0.05) were selected for mass spectrometry analysis.

#### Mass spectrometry identification of proteins and database search

Differentially expressed spots were excised from preparative gels, trypsin digested and analyzed by liquid chromatography-mass spectrometry (LC-MS/MS) as described in previous publications [23], [24]. The tryptic peptides extracted from the gel spots were analyzed using LC-MS/MS on a QTOF Ultra hybrid quad-TOF mass spectrometer (Waters/Micromass), following setup parameters as previously described [25].

Peak lists were generated using MASCOT Distiller (Matrix Science, London, England) and submitted to the database search program MASCOT (version 2.1 or 2.2, Matrix Science). Protein identification was achieved as described [24], by combining spectrum quality scoring obtained from a conventional database search program MASCOT (Version 2.1 or 2.2, Matrix Science, London, England). Search parameters were: peptide and MS/MS tolerances of 0.25 and 0.2 Da respectively, variable modifications were acrylamide, carbamidomethyl cys, met oxidation, peptide charge of 2+, 3+, and 4+, enzyme specificity was trypsin, one missed cleavage was allowed. NCBInr proteobacteria databases were searched (NCBInr 20131020, 33055681 sequences; 11532217697 residues).

#### Quantitative Reversed Transcribed PCR

A number of proteins identified in the proteomic analysis were selected for validation by qRT-PCR. Only those proteins of known function were selected, representing a variety of functional groups. *A. brasilense* specific primers for the genes of interest were designed using Primer3 (http://frodo.wi.mit.edu/). A list of primers used in this study is given in Table 2. The most stable reference genes (*GyrA* and *GlyA*) were selected, following a screen of 10 potential reference genes [26]. Total RNA was extracted from cell samples using a TRIzol Max Bacterial Isolation kit (Invitrogen, USA). cDNA was synthesized using in random hexamer primed reactions using a SuperScript III first strand synthesis kit (Invitrogen, USA). qRT-PCR reactions were carried out in a Rotor-Gene Q thermal cycler (Corbett Research, Australia). Each reaction contained 1×IQ SYBR Green Supermix (Bio-Rad), 0.5 µM each forward and reverse primer, and cDNA transcribed from 10 ng RNA. Samples from four independent experiments were analyzed in triplicate, and interplate and negative controls were included in each assay. Ct values were converted into expression data (relative to means of reference genes) using the Excel add-in Genex (Bio-Rad, USA). Statistical analysis was performed using GraphPad Prism software (GraphPad Software, USA). A students t-test (P<0.05) was used to determine statistically significant differences between group means.

## Results

### Sp7-flcAΔ is impaired in flocculation, Congo-Red binding and root colonization

In the *flcA* in-frame knock out strain Sp7-flcAΔ amino acid residues 16 to 212 of FlcA were replaced by a 1.5 kb fragment from pBR322 containing the tetracycline resistance gene. Sequencing of PCR-amplified DNA fragments containing the *flcA* region and Southern-blot analysis confirmed the successful knockout of the *flcA* gene in Sp7-flcAΔ (S1 Supporting Information). The tetracycline probe hybridized only to Hind III-digested Sp7-flcAΔ genomic DNA and to the plasmid pBR322 (containing the tetracycline resistance gene) and not to wild-type Sp7 genomic DNA, whereas, the *flcA* probe only hybridized to Hind III-digested Sp7 genomic DNA and to the plasmid pAB2062b (containing the *flcA* gene sequence) and not to Sp7-flcAΔ DNA. All positive hybridization bands were at the expected size (S1 Supporting Information).

**Figure 1:**
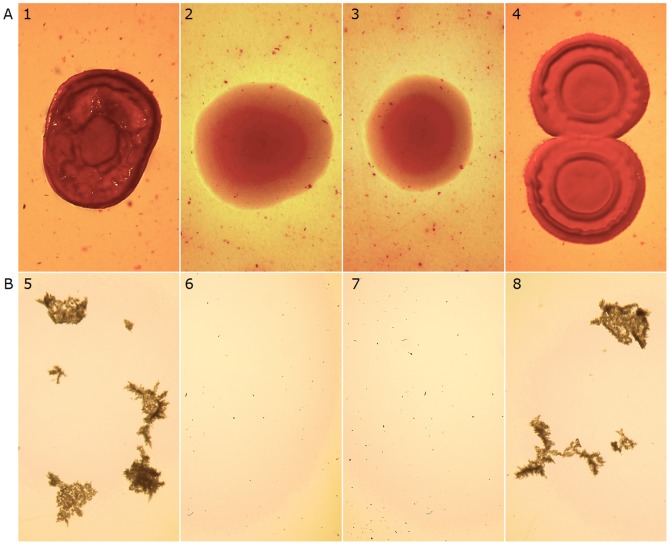
Congo-red binding and flocculation of *A. brasilense* strains. A. Wild type strain Sp7 (1) and *flcA* complemented strain Sp7-flcAΔ[pAB2053] (4) form dark red, rough surfaced colonies on NFM agar containing Congo-Red, whereas, Sp7-flcAΔ (2) and Sp72001 (3) form light-red, mucoid, smooth-surfaced colonies on the same medium. B. Flocculation of Sp7 (5) and Sp7-flcAΔ[pAB2053] (8) in flocculation liquid medium; Sp7-flcAΔ (6) and Sp72001 (7) do not flocculate under the same conditions.

Phenotypes controlled by FlcA, such as flocculation, Congo-Red binding and plant colonization [6] were impaired in *flcA* knock-out strain Sp7-flcAΔ. Wild-type Sp7 and Sp7-flcAΔ did not flocculate in nutrient medium. However, Sp7 cells started to flocculate within 3-4 hours when transferred to minimal medium supplied with a high ratio of fructose to KNO_3_, whereas Sp7-flcAΔ did not flocculate under these conditions (Fig. 1), consistent with our previously published work for *flcA* ^−^ mutant strain Sp72001 [6].

Congo-Red dye binds to bacterial specific lipopolysaccharides (LPS) and is commonly used in microbiological epidemiology for identification purposes [27]. When grown on agar media containing Congo-Red, the wild type strain Sp7 formed dark red and dry surfaced colonies, whereas Sp7-flcAΔ formed light red and smooth surfaced, mucoid colonies (Fig. 1). Complementation of Sp7-flcAΔ with the plasmid pLA2053 containing *flcA* restored wild-type phenotypes (Fig. 1), confirming that *flcA* deletion disabled Congo-Red binding by the Sp7-flcAΔ mutant cells.

**Figure 2:**
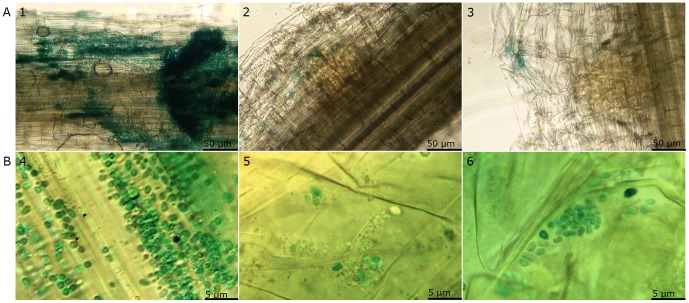
Plant-root binding abilities of *A. brasilense* strain. Colonization of wheat root by Sp7 (1 and 4), Sp7-flcAΔ (2 and 5) and Sp72001 (3 and 6). *A*. *brasilense* strains harbor the reporter plasmid pLA-lacZ, containing a constitutively expressed *lacZ* gene and were stained with X-gal. Sp7 (1, 4) has strong binding ability to wheat roots and can be found all over the root surface; Sp7-flcAΔ (2, 5) and Sp72001 (3, 6) lost the ability to bind to wheat roots and could only be found in lateral root emergence areas. Scale bars indicate 50 µm (group A) and 5 µm (group B) (Magnification ×100 in group A and ×1000 in group B).

As shown previously for *flcA* ^−^ mutant Sp72001 [6], Sp7-flcAΔ was also impaired in wheat root-surface colonization (Fig. 2) and *lacZ*-labeled Sp7-flcAΔ cells were detected by X-gal staining only in the crevices of lateral root emergence sites (Fig. 2). This is in contrast to the wild-type strain Sp7, which intensively colonized the surface of wheat roots.

Complementation with pAB2053, carrying the *flcA* gene, restored all phenotypes to Sp7-flcAΔ [pAB2053] (Fig. 1).

### Proteome and transcriptome analysis of wild-type Sp7 and Sp7-flcAΔ

**Figure 3:**
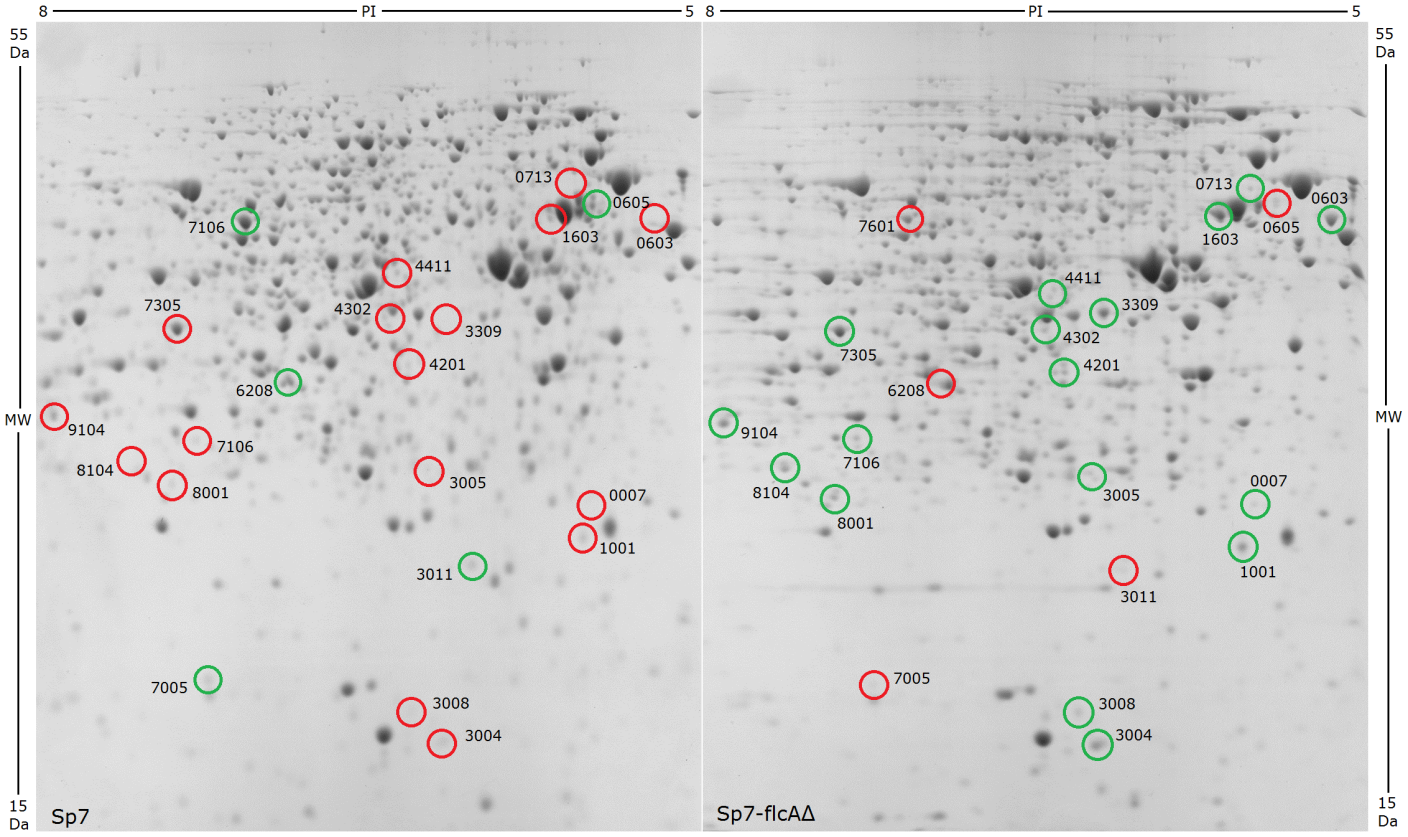
2-DE gels of *A. brasilense* Sp7 and Sp7-flcAΔ under flocculation conditions. Blue silver stained 2-DE gel of proteins extracted from *Azospirillum* Sp7 (A) and Sp7-flcAΔ mutant (B) under flocculation conditions. Circled spots were found to be differentially expressed and were able to be excised and analyzed by LC-MS/MS. Green circles indicate increased expression and red circles indicate decreased expression in either Sp7 or Sp7-flcAΔ.

Analysis of 2-DE gels showed reproducible protein patterns between sample replicates (intra-assay CV% = 9.97) and between independent experiments (inter-assay CV% = 25.61). Gel analysis by PDQuest advanced 2-D analysis software (Bio-Rad) revealed 33 protein spots with differential expression (2-fold changes (*P*<0.05) between Sp7 and Sp7-flcAΔ. Twenty two out of the 33 protein spots (Fig. 3) could be confidently manually excised for analysis by LC-MS/MS (Table 3). The other spots were either too small or located too close to other spots so that excision without cross-contamination would have been difficult. In order to provide as complete a description of our experiments as possible we have included the outcome of the 2DE-gel analysis in total. Representative gel images of typical 2-DE gels of A. *brasilense* Sp7 and Sp7-flcAΔ under flocculation conditions are shown in Fig. 3, with circles and numbers indicating differentially expressed protein spots. Note that to qualify for further LC-MS/MS analysis as a deregulated spot, inclusion criteria were that it must be expressed in all six replicate gels and have a significantly different expression level compared with the other six gel replicates of its counterpart sample. Additionally in some cases a spot was present only in one strain but not the other. In this all-or-nothing case statistical analysis could not be performed, but the spot was analyzed by LC-MS/MS whenever possible. The proteins identified in each spot that are homologous to *Azospirillum brasilense* Sp245 are listed in Table 3 and S2 Supporting Information. Some of the 2DE gel spots had several peptides listed with sequence homology to more than one protein. Although the 2-DE gels in this work were well resolved, co-migration of multiple proteins is possible, as shown previously by Lim et al. [28].

**Table 3:** Proteins regulated by FlcA.

Spot number	Up-regulated protein and accession number	Reference	Function	Score (emPAI)	Matches (% coverage)
0605	GatB, aspartyl/glutamyl-tRNA(Asn/Gln) amidotransferase subunit B, gi|392378551	[89]	Translation	1398 (2.31)	29 (50%)
3011	conserved protein of unknown function, gi|392377282	[89]	unknown	733 (15.70)	14 (52%)
6208	conserved protein of unknown function; putative nucleoside triphosphate hydrolase domain, gi|392379902	[89]	unknown	602 (1.30)	14 (46%)
	GalU, UTP-glucose-1-phosphate uridylyltransferase, gi|392380036	[89]	Carbohydrate metabolism	222 (0.34)	7 (25%)
7005	CheW, chemotaxis signal transduction protein CheW, gi|392378434	[89]	Signal transduction, Cell motility	565 (1.77)	14 (53%)
	maoC domain protein dehydratase, gi|392380146	[89]	unknown	185 (0.40)	5 (27%)
7601	GltA, citrate synthase (fragment), partial, gi|392382140	[89]	Carbon metabolism	498 (22.85)	31 (81%)

Spot number	Down-regulated protein and accession number		Function	Score (emPAI)	Matches (% coverage)
0007	conserved protein of unknown function; putative signal transduction histidine kinase domain, gi|392378431	[89]	unknown	791 (4.24)	18 (74%)
	*ClpP, ATP-dependent Clp protease proteolytic subunit, gi|194595677	[90]	Cell growth and death	189 (0.31)	6 (30%)
0603	AtpD, ATP synthase, F1 sector, beta subunit, gi|392381407	[89]	Energy metabolism	1463 (4.85)	26 (73%)
0713	PuuA, gamma-Glu-putrescine synthase, gi|392382441	[89]	Amino acid metabolism	860 (0.89)	22 (46%)
1001	AhpC, alkyl hydroperoxide reductase, C subunit, gi|392378525	[89]	Oxidoreductases	421 (10.30)	19 (63)
1603	GlnA, glutamine synthetase, gi|392382076	[89]	Amino acid metabolism	638 (4.57)	36 (74%)
3004	conserved protein of unknown function; CBS domain, gi|392380710	[89]	Unknown	527 (4.71)	10 (59%)
	Glo1, glyoxalase I, gi|392382135	[89]	Carbohydrate metabolism	450 (0.85)	12 (52%)
	RplT, 50S ribosomal subunit protein L20, gi|392380695	[89]	Translation	248 (0.54)	6 (47%)
	conserved protein of unknown function, gi|392380531	[89]	unknown	196 (1.23)	5 (55%)
	FabA, Dehydratase, gi|194595687	[90]	Lipid metabolism	138 (0.75)	3 (22%)
3005	PyrE, orotate phosphoribosyltransferase, gi|392381037	[89]	Nucleotide metabolism	969 (6.62)	22 (53%)
	conserved hypothetical protein; putative proteasome-type protease domain, gi|392383793	[89]	unknown	697 (2.18)	14 (46%)
	putative Cyclohexadienyl dehydratase precursor [Includes: Prephenate dehydratase; Arogenate dehydratase], gi|392379712	[89]	unknown	531 (0.88)	10 (43%)
3008	putative acyl dehydratase, gi|392381647	[89]	unknown	247 (2.20)	5 (26%)
	PhhB, 4-alpha-hydroxy-tetrahydropterin dehydratase (fragment), partial, gi|392381718	[89]	Lyases	198 (3.11)	5 (46%)
3309	putative CDP-tyvelose-2-epimerase, gi|392383820	[89]	unknown	1309 (4.81)	27 (71%)
	putative NH(3)-dependent NAD(+) synthetase, gi|392378273	[89]	unknown	670 (1.79)	14 (50%)
4201	putative ABC transporter, substrate-binding component, gi|392382197	[89]	unknown	1464 (6.59)	26 (76%)
	RplY, 50S ribosomal protein L25, gi|392380061	[89]	Translation	231 (0.76)	6 (38%)
4302	putative NH(3)-dependent NAD(+) synthetase, gi|392378273	[89]	unknown	682 (14.46)	31 (71%)
	PotD, putrescine ABC transporter, periplasmic binding protein, gi|392382440	[89]	Membrane transport	439 (2.97)	21 (51%)
4411	putative ATPase (MoxR-like), gi|392378561	[89]	unknown	1228 (5.36)	22 (62%)
	LivK, branched-chain amino acid ABC transporter, substrate-binding periplasmic component, gi|392382325	[89]	Membrane transport	549 (1.25)	12 (37%)
	*PhaA, acetyl-CoA acetyltransferase with thiolase domain (Beta-ketothiolase), gi|392378463	[89]	Carbon metabolism	381 (0.49)	7 (23%)
7106	HisG, ATP-phosphoribosyltransferase, gi|392378314	[89]	Amino acid metabolism	746 (2.30)	16 (58%)
	putative hydroxyacid oxidoreductase (Fe-S centre), gi|392380858	[89]	unknown	403 (0.77)	10 (44%)
	NalL, nitrate/nitrite response regulator protein NarL, gi|392380319	[89]	Intracellular signal transduction	318 (0.47)	9 (44%)
	putative dioxygenase, gi|392381692	[89]	unknown	300 (0.57)	7 (17%)
	LpxA, UDP-N-acetylglucosamine acyltransferase, gi|392382132	[89]	Glycan biosynthesis and metabolism	192 (0.57)	4 (20%)
	putative Cyclohexadienyl dehydratase precursor [Includes: Prephenate dehydratase; Arogenate dehydratase], gi|392379712	[89]	unknown	173 (0.23)	4 (20%)
	PqqC, coenzyme PQQ (pyrroloquinoline quinone) synthesis protein C, gi|392382168	[89]	Oxidoreductase	133 (0.38)	3 (17%)
7305	NAD-dependent epimerase/dehydratase, gi|392383823	[89]	Carbohydrate metabolism	1049 (2.85)	23 (58%)
	GapA, glyceraldehyde-3-phosphate dehydrogenase (GAPDH), gi|392382782	[89]	Carbon metabolism, Energy metabolism	388 (0.55)	10 (31%)
8001	NOS, nitric oxide synthase, gi|210062910	[91]	Amino acid metabolism	550 (1.42)	15 (51%)
	SodA, superoxide dismutase, gi|392379921	[89]	Oxidoreductases	504 (3.47)	11 (43%)
8104	protein of unknown function; putative response regulator domain, gi|392382762	[89]	unknown	766 (2.47)	17 (51%)
	putative phosphate starvation-inducible protein with ATPase activity, PhoH-like, gi|392381702	[89]	unknown	506 (1.57)	13 (74%)
	putative 3-oxoacyl-(acyl-carrier-protein) reductase, gi|392381311	[89]	unknown	230 (0.27)	6 (22%)
9104	NarL1, two-component transcriptional regulator (with NarX) of nitrate reductase, gi|392383223	[89]	Intracellular signal transduction	836 (8.77)	18 (67%)
	conserved protein of unknown function; putative cysteine hydrolases domain, gi|392380057	[89]	unknown	531 (0.99)	14 (42%)

*these two proteins marginally missed the emPAI criteria that we have applied for inclusion in this table (i.e., emPAI≥10% of the highest abundant protein in each spot), however they were identified in the mascot search and confirmed as downregulated using qRT-PCR (see Fig. 5). Functions were according to Kyoto Encyclopedia of Genes and Genomes (KEGG, http://www.genome.jp/kegg/). Full details of peptide sequences and mascot search output on which this summary table is based are provided in S2 Supporting Information.

**Figure 4:**
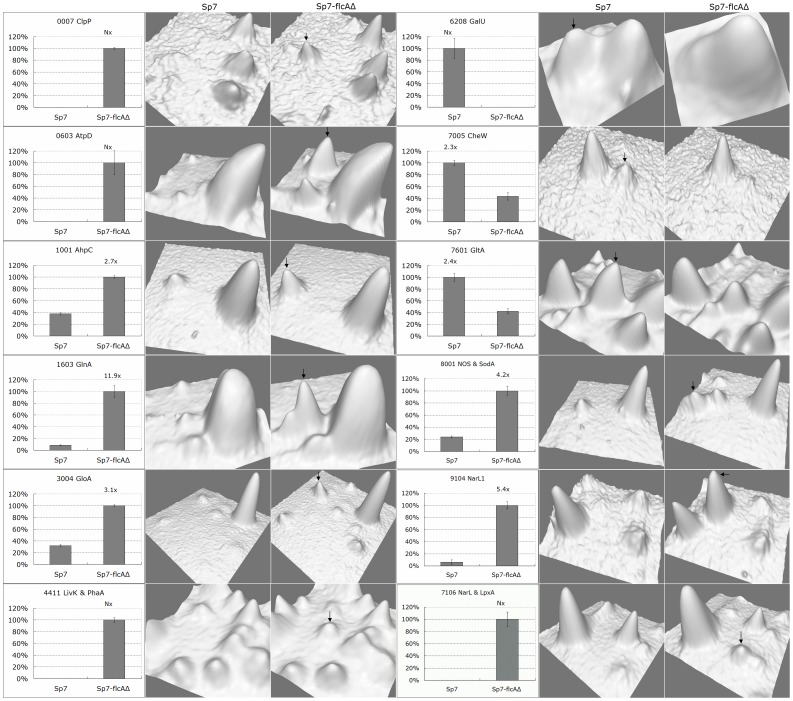
Differential protein expression in Sp7 and Sp7-flcAΔ analyzed by 2-DE. The left section shows the normalized expression volume of the spot in wild type (Sp7) and *flcA* knock out strain (Sp7-flcAΔ) under flocculation conditions; the relative fold change is shown above each column (Nx indicates that relative fold change could not be calculated as the protein was only detected in either Sp7 or Sp7-flcAΔ). The right section is a 3D representation of the area of interest as provided by PDquest software. Arrows indicate spots with relatively higher expression.

**Figure 5:**
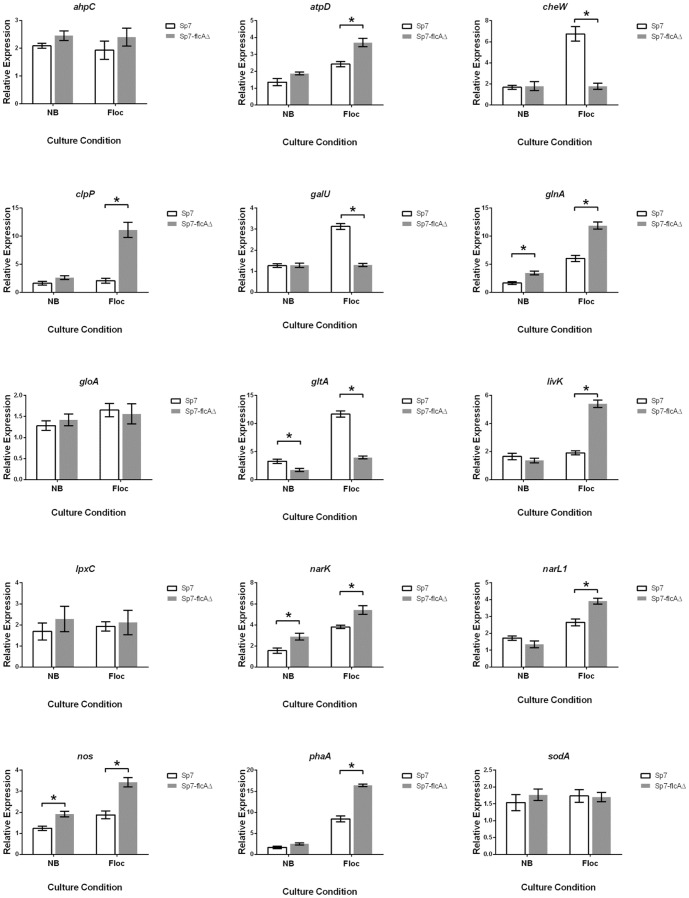
Differential gene expression in Sp7 and Sp7-flcAΔ under both nutrient and flocculation conditions, analyzed by qRT-PCR. Transcription levels of *atpD, clpP, glnA, livK, nark, narL1, nos* and *phaA* were significantly higher in Sp7-flcAΔ than in Sp7 under flocculation conditions. Transcription levels of *cheW, galU* and *gltA* were significantly lower in Sp7-flcAΔ than in Sp7 under flocculation conditions. Transcription levels of *ahpC, gloA*, *lpxC* and *sodA* did not differ significantly in Sp7 and Sp7-flcAΔ under either culture condition. * indicates a significant difference between group means (P<0.05). Data is presented as mean ±SEM, relative to expression of two reference genes, n = 4 biological replicates.

Five of the excised protein spots had higher expression levels in wild-type Sp7 than in *flcA* deletion strain Sp7-flcAΔ, indicating that these proteins were up-regulated in the presence of FlcA (Table 3; Fig. 4). Three of these proteins, (UTP-glucose-1-phosphate uridylyltransferase (GalU), chemotaxis signal transduction protein (CheW) and citrate synthase (GltA)) were selected for further analysis by qRT-PCR, which confirmed an increase in *galU, cheW* and *gltA* expression in wild-type Sp7 compared to Sp7-flcAΔ under flocculation conditions (Fig. 5).

Seventeen of the excised protein spots showed lower expression levels in Sp7 compared to Sp7-flcAΔ, indicating that these proteins were down-regulated in the presence of FlcA (Table 3; Fig. 4). Ten of these proteins were selected for further analysis by qRT-PCR, which confirmed increased expression in Sp7-flcAΔ of ATP-dependent Clp protease proteolytic subunit (*clpP*), nitric oxide synthase (*nos*), branched-chain amino acid ABC transporter (*livK*), glutamine synthetase (*glnA*), ATP synthase F1 sector beta subunit (*atpD*) and beta-ketothiolase (*phaA*). No difference in mRNA levels was observed for alkyl hydroperoxidase (*ahpC*), glyoxalase 1 (*gloA*) or superoxide dismutase (*sodA*) (Fig. 5).

Additional genes, reported by Valverde et al. [12] to be differentially expressed in Sp7 and *flcA* ^−^ mutants, were also analyzed by qRT-PCR. There was no significant difference in expression of UDP-3-O-acyl-N-acetylglucosamine deacetylase (*lpxC*) between Sp7 and Sp7-flcAΔ under flocculation conditions (Fig. 5). Analysis of the nitrate/nitrite transporter (*narK)* expression revealed an increase in expression in Sp7-flcAΔ compared to the wild-type Sp7 under both nutrient and flocculation conditions (Fig. 5).

## Discussion

The regulatory gene *flcA* of *Azospirillum brasilense* Sp7 controls processes that are involved in cell morphological transformation from motile vegetative to immobile, cyst-like, EPS-coated cells. It thus regulates the ability of the cells to flocculate and to adhere to roots and interact with plants [6]. The lack of flocculation, reduced Congo-Red binding and lack of binding to roots by the *flcA* deletion mutant Sp7-flcAΔ confirmed the role of *flcA* in these processes. The results presented here provide the molecular evidence for FlcA involvement in stress tolerance, carbohydrate metabolism, cellular transformation and nitrogen fixation.

### Stress tolerance

Alkyl hydroperoxidase (AhpC), functions as a bacterial chaperone protein, participating in antioxidant defense against H_2_O_2_-induced stress in *Escherichia coli* and UV-light induced oxidative stress in *Salmonella typhimurium* [29]–[31]. It also participates in the response to hyperosmotic shock in *Staphylococcus aureus* [32] and to heat-shock in *Escherichia coli* O157:H7 and *Myxococcus xanthus* [33], [34]. In *A. brasilense* Sp245 AhpC is involved in resisting peroxide stress, and has also been implicated in stress responses to nutrient limiting conditions [35]. A defect in AhpC function impairs the ability of cells to aggregate and flocculate under nutrient-limiting conditions [35]. Since both FlcA and AhpC are required for flocculation under stressful situations it is unlikely that FlcA negatively regulates the expression of *ahpC* as the increase in AhpC expression in the absence of FlcA may suggest. It is more likely that FlcA is indirectly involved in regulating the cellular response to stress; the cells lacking *flcA* cannot flocculate but are still exposed to stress whereas the wild type cells flocculate and avoid the stress.

ATP-dependent Clp protease proteolytic subunit (ClpP) complexes are essential for virulence and survival under stress and starvation conditions in bacteria [36]–[38]. Moreover, protease complexes participate in overall proteolysis of misfolded proteins generated under stress conditions and starvation in bacteria [39]–[43]. Inactivation of ClpP leads to the up-regulation and accumulation of stress-proteins, mainly oxidative stress proteins, in *S. aureus*, indicating that ClpP plays an important role in maintaining protein homeostasis [44]. The lack of morphological transformation of Sp7-flcAΔ into stress-tolerant cyst-like forms may have resulted in more misfolded proteins accumulating in the stress-liable cells in the absence of functional FlcA and thus over-expression of *clpP*.

Both nitric oxide synthase (Nos) and superoxide dismutase (SOD) have been implicated in the oxidative stress response in various bacterial species. Nitric oxide, produced by NoS, is a highly reactive free radical shown to protect bacterial species against both oxidative and antibiotic-induced oxidative stress [45], [46]. SODA functions in the cellular detoxification of superoxides [47]. Expression of SODA helps to protect bacterial cells from oxidative stress, while cells lacking SODA show increased sensitivity to oxygen species [48], [49]. In this study both NoS and SODA were found to be more highly expressed in the flocculation impaired Sp7-flcA knockout than in wild-type Sp7, indicating that FlcA-deficient cells may be affected in the expression of stress response genes, including those involved in oxidative stress response.

Taken together these results suggest that FlcA has a role in multiple responses to various stress conditions, as its presence is required for morphological transformation into cyst-like cells with greater stress resilience. Nutritional stress under flocculation conditions is aggravated in Sp7-flcAΔ as the cells are unable to fully respond to this stress by normal transformation into cyst-like, relatively dormant forms. Indeed, *Azospirillum* mutants affected in EPS production and aggregation show reduction in stress tolerance [50]. The inability of Sp7-flcAΔ cells to undergo flocculation results in prolonged exposure to stressful conditions, and promotes the expression of stress response genes. Further studies are required for full understanding of the role of FlcA in stress endurance in *Azospirillum*.

### Production of polysaccharides and poly-β-hydroxybutyrate

Citrate synthase (GltA) is an enzyme in the tricarboxylic acid (TCA) cycle, utilizing acetyl-CoA as a substrate. Synthesis of intracellular PHB storage granules from acetyl-CoA can act as an overflow pathway from the TCA cycle [51]. The synthesis and utilization of PHBs under stress conditions favors the survival of *A. brasilense*, with PHB-accumulating cells exhibiting increased stress endurance [52], [53]. PHB is synthesized initially from two acetyl-CoAs to form acetoacetyl-CoA by beta-ketothiolase (PhaA) [9], [54]. Based on the observations that PhaA is overexpressed in Sp7-flcAΔ, whereas, GltA is overexpressed in Sp7, we suggest that these two enzymes, which compete for the same substrate [55], are reciprocally regulated under flocculation conditions. It seems that under prolonged stress conditions, the absence of FlcA in flocculation-impaired cells results in PHB synthesis taking place preferentially over citric acid cycle metabolism. Previous studies have suggested that PHB is present in *flcA* ^−^ mutant cells [11], however the data presented were qualitative and did not compare the quantities of the PHB in the presence and absence of FlcA. Further studies are required to determine whether FlcA directly regulates the expression of these enzymes.

GltA has also been implicated in EPS production, as a defect in *gltA* alters cell surface polysaccharides of *Sinorhizobium meliloti*. The growth of *gltA* ^−^ strains is relatively normal if a source of glutamate is available, but a modification of EPS composition was detected [49]. The role of FlcA in the production of intact and complete EPS and capsular material in *Azospirillum* is well established both morphologically [6] and chemically [7], [8]. One of the mechanisms by which FlcA influences EPS composition may be by activating GltA, altering the flow through the TCA cycle and promoting complete sugar assimilation into the EPS.

UTP-glucose-1-phosphate uridylyltransferase (GalU) catalyzes the formation of UDP-glucose, which is involved in synthesis of glycosylated surface structures, and in the enzymatic biosynthesis of carbohydrates [56]. Mutations in the *galU* gene have led to reduced virulence in a number of pathogenic bacteria, attributed to changes in LPS and EPS production [57]–[59]. *GalU* mutants of *Streptococcus pneumoniae* are unable to synthesize capsule polysaccharides [60], and *galU* mutants of *E. coli* have been shown to be defective in surface adhesion [61]. The overexpression of GalU in wild-type Sp7 compared to Sp7-flcAΔ suggests that FlcA may regulate EPS production through the regulation of GalU.

UDP-N-acetylglucosamine acyltransferase (LpxA) and UDP-3-O-(R-3-hydroxymyristoyl)-GlcNAc deacetylase (LpxC) catalyze the first two steps of the biosynthetic pathway of Lipid A, a component of the outer membrane lipopolysaccharide in Gram-negative bacteria [62], [63]. In *Escherichia coli*, LpxC is elevated approximately 5-fold when lipid A synthesis is inhibited [64]. In this study, LpxA was found over-expressed in Sp7-flcAΔ under aggregated conditions, in which the outer membrane materials were defective, suggesting that FlcA is involved in biosynthesis of outer membrane lipopolysaccharide.

### Morphological transformation and plant root colonization in *A. brasilense*

Chemotaxis allows motile bacteria to sense and adapt to a changing environment by allowing movement towards more favorable environmental conditions. Chemotaxis in *A. brasilense* is controlled by the Che1 pathway, which comprises homologues of CheA, CheW, CheY, CheB, and CheR. The genes involved in the *A. brasilense* Che1 pathway not only regulate taxis behaviors but are also involved in other cellular functions including cell-to-cell clumping and flocculation [65], [66]. Transducer coupling protein (CheW) has a very well demonstrated role in flagella biosynthesis and movement control of bacteria, but homologous genes have also been implicated in cyst development. A mutant of a CheW-homologous gene in *Rhodospirillum centenum* failed to form cyst cells in response to starvation [67] and a mutation in a CheW-homologue in *Myxococcus xanthas* resulted in defects in developmental aggregation, sporulation, and motility [68]. In previous studies, it was found that *flcA* mutants of *A. brasilense* are unable to undergo transition from vegetative into non-motile encapsulated cyst-like forms and remain motile [6], [11], [13]. Its reduced expression in Sp7-flcAΔ under flocculation conditions emphasized the involvement of the CheW-homologous gene in the transformation of *Azospirillum* from vegetative to cyst-like cells.

### Nitrogen metabolism

In a previous study, *flcA* mutants were found to have higher nitrogenase activity than wild-type Sp7 when in association with plants, and this was attributed to their ability to remain in the vegetative state on the roots [11]. Interestingly, glutamine synthetase (GlnA), the main subunit of the glutamine synthetase complex, is up-regulated in Sp7-flcAΔ. GlnA catalyzes the reaction: Glutamate + NH_3_ + ATP → Glutamine + ADP + Pi, which plays an important role in nitrogen assimilation in *Azospirillum* and thus also indirectly in the regulation of enzymes involved in nitrogen fixation, such as nitrogenase [69]–[71]. Higher expression of *glnA* in flocculation medium than in nutrient medium, in both wild-type and *flcA* ^−^ mutant strains, is in agreement with previous studies showing that in *A. brasilense glnA* is transcribed at high levels under nitrogen limited conditions, and at lower levels in the presence of excess nitrogen [72], [73]. The observation that *glnA* was up-regulated in Sp7-flcAΔ mutant cells suggests that FlcA may control nitrogen assimilation by down-regulating glutamine synthetase in the wild-type, and thus affecting cellular ammonium concentrations in *Azospirillum*.

NarX-NarL and NarQ-NarP are pairs of two-component regulatory systems that control *Escherichia coli* gene expression in response to the respiratory oxidants nitrate and nitrite [74]. Nitrate stimulates the autophosphorylation rates of the NarX and NarQ sensors, which then phosphorylate the response regulators NarL and NarP to activate and repress target operon transcription [75], [76]. In this study, NarL and its homologue NarL1 were both found to be down-regulated in the presence of FlcA when a high C:N ratio medium was applied and nitrate was the sole source of nitrogen. This is in agreement with the previous study where *flcA* mutants were found to have higher nitrogenase activity than wild-type Sp7 when in association with plants [11]. The results suggested that FlcA is involved in nitrate assimilation in a NarX-NarL dependent manner.

### FlcA target genes: two complementary studies

In another study, published by Valverde et al. [12], FlcA target genes were investigated using nucleic acid based techniques. In both studies FlcA has been implicated in carbon and nitrogen metabolism, however, each study revealed a different set of genes/proteins involved in FlcA-controlled cellular processes occurring during flocculation.

FlcA control was studied at the mRNA level by cDNA-AFLP in Valverde's work [12], whereas here it was studied at the protein level by 2-DE and using qRT-PCR to measure gene expression, making the two studies complementary rather than identical. Both studies found that FlcA was involved in carbon reserve and metabolism. A member of MotA/TolQ/ExbB proton channel [77] family was found up-regulated by FlcA in Valverde's work [12], suggesting that FlcA could mediate the development of the outer coat of EPS and/or other biopolymers by regulating transporter complexes similar to that of *Sphingomonas sp.* A1 [12], [77]. In this study, GltA and PhaA were found to be controlled by FlcA, suggesting that FlcA is involved in channeling acetyl-CoA between TCA cycle and PHB synthesis/degradation cycle in *Azospirillum*.

It is worth mentioning that a transcript-derived fragment (TDF) homologue (AZ79) to UDP-3-O-acyl-N-acetylglucosamine deacetylase (LpxC) was found up-regulated by FlcA under flocculation conditions in *A. brasilense* Sp7 [12]. In this study, we were unable to detect a significant difference in *lpxC* expression between Sp7 and Sp7-flcAΔ under flocculation conditions. However, LpxC is the second enzymatic step in LPS biosynthesis, catalyzing a deacetylation step of UDP-3-O-(R-3-hydroxytetradecanoyl)-GlcNAc [78], [79]. Thus, it follows that LpxC be regulated by FlcA as *flcA* ^−^ mutants were shown to be weakly stained by Congo-Red when compared with wild-type Sp7, suggesting a defect in their LPS production [6]. Notably, the relationship between FlcA and LpxC may have important biomedical significance, as LpxC is crucial for the survival of Gram-negative bacteria and has no sequence homology to known mammalian deacetylases or amidases, thus, it is a potential target for the design of new antibiotics [80].

Both studies found that FlcA was involved in nitrogen metabolism. In Valverde et al. [12] a nitrate/nitrite transporter (NarK), belonging to the major facilitator superfamily of transmembrane transporters [81]–[83] was found down regulated by FlcA, suggesting the involvement of FlcA in nitrate/nitrite transport in *A. brasilense* [11]. In this study, NarL and NarL1, the transcriptional activators of Nark [84], were also found to be up-regulated by FlcA. The increase in *narK* and *narL1* expression in Sp7-flcAΔ compared to the wild-type sp7 under both nutrient and flocculation conditions and the down-regulation of glutamine synthetase by FlcA, suggest that FlcA may control nitrogen assimilation and nitrogen fixation by down-regulating glutamine synthetase in *Azospirillum*.

The only common target of FlcA found in both studies is AtpD (F0F1 ATP synthase). However, Valverde et al. [12] found AtpD to be up regulated by FlcA whereas here it was found to be down regulated by FlcA, at both protein and gene expression levels under flocculation conditions. Since cells were collected before flocculation began in Valverde et al. [12] and after flocculation in the current study, it is possible that metabolism has substantially changed during formation of visible aggregates. Wild-type Sp7 and *flcA* ^−^ Tn5-induced mutant Sp72002 were used by Valverde et al. [12], whereas Sp7 and Sp7-flcAΔ were used in this study. Another reason for the difference in the list of candidates of FlcA-regulated genes/proteins between the two studies may be the differences in the techniques used and their limitations.

## Conclusions

This study has demonstrated the usefulness of the proteomic and transcriptomic approach to identify proteins involved in FlcA-mediated flocculation in *Azospirillum brasilense*. Groups of proteins associated with altered FlcA expression were identified, including proteins involved in stress responses, morphological transformation and nitrogen fixation. The protein patterns discovered in this study clarify current knowledge on the phenotypes observed in *flcA* knock out or Tn5 induced mutant strains. Further analysis is required to determine whether these proteins are regulated directly by FlcA, or whether they are controlled by FlcA mediated processes. Those directly controlled by FlcA may be identified by searching for conserved helix-turn-helix binding motifs upstream of the genes encoding such differentially expressed proteins. Moreover, further time-course based proteomic investigations during the flocculation process and determination of target genes using direct methods, such as gel shift assays, will provide a more comprehensive view of FlcA target genes/proteins in *A. brasilense*. Through the identification of proteins and genes involved in flocculation, this study enhanced the knowledge of cellular responses of *Azospirillum* during morphological transformation.

## Supporting Information

S1 Supporting Information **Southern blot analysis of Sp7-flcAΔ knockout strain. (**A) Structures of the *flcA* regions in three strains are illustrated by colored bars, *flcA* in white, *tet* and its promoter in grey and Tn5 insertion in black (not to scale). Arrow indicates the *flcA*-upstream region in Sp72001, where the *flcA* gene is disrupted by Tn5 insertion. Insertion of the tetracycline gene and *flcA* knockout was confirmed by Southern Blot hybridization of HindIII digested genomic DNA. (B) DIG labeled *tet* probe was applied; lane 1, Sp7 wild-type genomic DNA; lane 2, linearized plasmid pAB2062b, carrying *flcA* gene, as a negative control; lane 3, Sp7-flcAΔ genomic DNA; lane 4, pBRd322 plasmid, the origin of the cloned tetracycline gene, as a positive control. (C) DIG labeled *flcA* probe was applied; lane 1, pAB2062b as a positive control; lane 2, Sp7 wild-type genomic DNA; lane 3, pBRd322 as a negative control; lane 4, Sp7-flcAΔ genomic DNA.
(TIFF)

S2 Supporting Information **Mascot search output for identification of protein spots.** Detailed mascot search output when NCBInr 20131020 database was searched using spectra from the LC-MS/MS data generated using tryptic digests of each of the 2D gel spots. This is an extension of the data summarized in Table 3 of the manuscript, and details the peptide sequences on which each of the protein identifications are based. Each of the proteins represented here are from *Azospirillum brasilense*.
(XLS)
